# A Case of Infectious Laryngotracheitis in an Organic Broiler Chicken Farm in Greece

**DOI:** 10.3390/vetsci8040064

**Published:** 2021-04-16

**Authors:** Vasileios Tsiouris, Natalia Mavromati, Konstantinos Kiskinis, Tilemachos Mantzios, Zalan G. Homonnay, Tamas Mato, Mihaly Albert, Istvan Kiss, Ioanna Georgopoulou

**Affiliations:** 1 Unit of Avian Medicine, Clinic of Farm Animals, Faculty of Veterinary Medicine, School of Health Sciences, Aristotle University of Thessaloniki, Staurou Voutyra 11, 54627 Thessaloniki, Greece; nataliem@vet.auth.gr (N.M.); kiskinik@vet.auth.gr (K.K.); mantzios@vet.auth.gr (T.M.); ioannag@vet.auth.gr (I.G.); 2 Ceva-Phylaxia, Szállás u. 5., 1107 Budapest, Hungary; zalan.homonnay@ceva.com (Z.G.H.); tamas.mato@ceva.com (T.M.); mihaly.albert@ceva.com (M.A.); istvan.kiss@ceva.com (I.K.)

**Keywords:** broiler chicken, infectious laryngotracheitis, organic farm, vaccinal strain, wild strain

## Abstract

Infectious laryngotracheitis is an economically significant viral disease of chickens, that mainly affects the upper respiratory tract, and is present worldwide. This case reports the first outbreak of infectious laryngotracheitis in a four-week-old organic broiler farm and surrounding flocks in Greece, with typical clinical symptoms and lesions, allegedly provoked by a wild strain of infectious laryngotracheitis virus. Our findings contradict the general perception indicating that the disease appears mainly in older birds and that vaccine strains are the primary cause of infectious laryngotracheitis outbreaks in most continents. A recombinant vectored vaccine was administered, supplementary to biosecurity measures, containing the viral spread. The responsible strain was potentially circulating in the area; therefore, an industry-wide holistic approach was applied, including the vaccination of neighboring broilers and breeders with the same vaccine, the rapid molecular diagnosis of the disease, and strict biosecurity protocols. The results of this holistic effort were effective because, following the application of vaccine and management protocols, manifestations of the disease in regional flocks dropped significantly, and there was no recurrence to date. These findings suggest that vaccination protocols should be modified, especially for organic broilers, to include vaccination against infectious laryngotracheitis.

## 1. Introduction

Infectious laryngotracheitis (ILT) is an acute, highly contagious, viral disease of poultry, primarily affecting the upper respiratory tract. ILT virus (ILTV) belongs to the species *Gallid alphaherpesvirus 1* and causes disease of great economic importance to the poultry industry, due to morbidity, mortality, weight loss, and decreased egg production, but the actual cost has not been determined [1]. The virus mainly affects chickens as a primary host, although infections can occur in pheasants, peacocks, turkeys, and guinea fowl. There is no proven transmission to humans or other mammals [2].

The direct transmission of ILT is completed horizontally via respiratory secretions, feces, and dust, whilst the exposure of birds to infected carriers and fomites, equipment, litter, and personnel contributes to the indirect transmission of ILTV. Recovered birds become long-term carriers of the virus, which enters a latent state, mainly in the trigeminal ganglia. Reactivation can occur after exposure to a stress factor, such as the onset of laying or moving of the birds, and carriers begin to shed the virus, infecting naïve birds [3,4].

Chickens of all ages can be affected, although ILT in field conditions usually concerns those from three to nine months of age [5], with adult birds displaying the most characteristic signs [6] (pp. 267–271). The disease presents either in a mild or a severe form and is regarded as a slow spreading disease in poultry flocks, depending on the type of housing system. The mild manifestation, with nasal discharge, respiratory rales, conjunctivitis, and little to no mortality, is similar to that of other respiratory pathogens. The clinical signs of the severe form include dyspnea, coughing of bloody mucus, along with periocular sinusitis and severe conjunctivitis. Layer hens demonstrate a decrease in egg production, while the growth of young poultry is affected [7,8,9]. Gross lesions in severe forms of the disease include the inflammation of the tracheal mucosa, with varying degrees of hemorrhage present, and the occurrence of mucoid or blood casts, caused by diphtheritic changes, located mainly in the larynx and the upper part of the trachea. Inflammation may affect and extend to the lungs and the air sacs. Likewise, ILTV causes swelling and congestion of the infraorbital sinuses and the conjunctiva epithelium [2,7]. The morbidity is high with levels from 50 to 100%, with the mortality levels varying from 0.1% in mild cases to 70% in severe outbreaks, depending on the virus strain, the viral load, the concurrent diseases, and the health status of the flock, among other factors [10,11].

Diagnosis relying solely on clinical signs and lesions is not possible; thus, it requires laboratory confirmation. Histopathology is the gold standard diagnostic method and is also a rapid diagnostic tool, because intranuclear inclusion bodies found in the tracheal and conjunctival epithelial cells are pathognomonic. However, it is worth mentioning that they are present only during the early stages of the infection [7,12]. Molecular diagnostic methods contribute to the definite conclusion and confirmation, with real-time polymerase chain reaction (PCR) superseding conventional PCR techniques during recent years [13]. Specific real-time PCR has been developed for the rapid detection of ILT, as well as thymidine kinase (TK) and glycoprotein G (gG) specific PCR assays, for sequence-based strain identification [14,15,16,17]. Furthermore, the virus can be isolated in embryonated chicken eggs or tissue cultures and detected by fluorescent antibodies or immunohistochemistry staining, thus additionally contributing to the diagnosis. Wild strains can be differentiated from vaccinal with the implementation of restriction fragment length polymorphism (RFLP) and genome sequencing [2,3,8,17,18].

The application of strict biosecurity measures is necessary for the prevention of the disease and attenuated or recombinant vectored vaccines are common for controlling the disease in endemic regions [4]. The first commercial vaccines introduced to the poultry industry were the live attenuated chicken embryo origin (CEO) vaccines around the late 1950s. They can introduce sufficient protection to the flock via eye drops, or through mass vaccination via the drinking water or aerosol spray, but can revert to their virulence, spread horizontally, and create latent carriers. Live attenuated tissue culture origin (TCO) vaccines, introduced in the late 1970s, are administered only via eye drop and have a lower ability of reversion to virulence, but their efficacy is lower compared to the CEO vaccines [19,20,21]. Both live attenuated vaccines have been held accountable for the majority of contemporary field outbreaks in many countries around the globe, thus introducing the term “vaccinal laryngotracheitis” [2,4]. Since the introduction of recombinant viral vector vaccines in the 2000s in the United States, their administration is now practiced worldwide, independently or in combination with live attenuated vaccines. They outmatch the side effects of live vaccines because they are reliable, do not create latent carriers, and do not regain virulence. However, they do not have the full potential in decreasing the replication and shedding of the virus [17,22].

With regard to the distribution of the disease, several reports indicate its worldwide spread [5,11,12,17,23,24,25]. Current reports are focusing on the emergence of vaccinal virus strains as the dominant cause of the outbreaks [3,9,16,26,27,28,29]. In the case of Greece, the country is practicing a general surveillance program for domestic poultry, according to the World Organisation for Animal Health [30]. The last references concerned two severe epizootic forms in the central part of the country in 1965 and 1971, possibly originating from a vaccinal strain administered via the vent in laying hens [31]. The objective of this report is to describe a field case of ILT in an organic broiler chicken farm in Greece, as well as to epidemiologically investigate the origin of the ILT strain by its sequence analysis and genetic characterization.

## 2. Case History

### 2.1. Flock History

The ILT outbreak was observed in an organic broiler chickens farm in the winter of 2015. The flock consisted of about 5000 chickens, originating from the same parent stock. The farm is located in the region of Epirus, Greece, an area characterized by large and very dense poultry populations and intensive poultry production, with records of the past decade indicating that the area concentrates 66.2% of total Greek broiler breeder farms and 45% of broiler farms [32]. It is worth mentioning that in the same region there were broiler, broiler breeder, layer, and backyard flocks, as well as a lake, conserving more than 170 species of birds (gulls, ducks, swans, etc.). Additionally, the reported farm resided in a plain, where composted manure from the local farms was discarded.

The vaccination program of the flock included vaccines against Marek’s disease virus (MDV), Newcastle disease virus (NDV), infectious bronchitis virus (IBV), and infectious bursal disease virus (IBDV). The neighboring broiler breeder flocks did not use vaccines against ILT, while the vaccination program of the layer hen farms of the area included a vaccination with the Nobilis^®^ ILT (MSD Animal Health, Hertfordshire EN11 9BU, London, UK), a live attenuated vaccine which was applied via eye drop.

### 2.2. Clinical Manifestation

**Figure 1:**
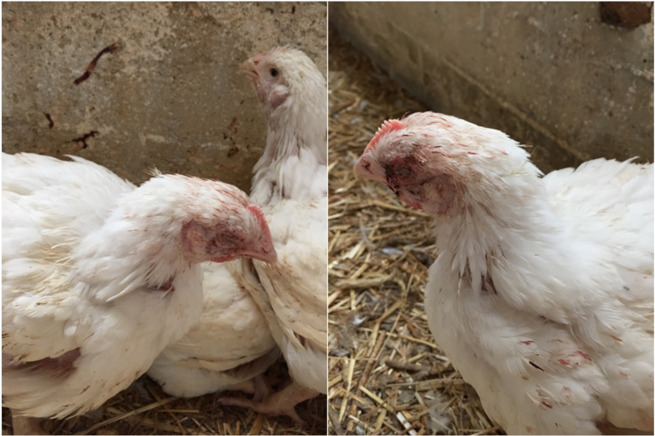
A 33-day-old broiler chicken with typical symptoms of ILT. In particular, bloody mucoid ocular exudate which soiled both the plumage (**right**) and the walls of the poultry house (**left**).

At 25 days of age, numerous broilers displayed respiratory symptoms; signs of dyspnea, rales, gasping, and expectoration of bloody mucus, along with depression and anorexia. Birds had developed conjunctivitis with ocular secretion, nasal discharge, and swelling of the infraorbital sinuses, as depicted in Figure 1. The farmer’s concern arose from the fact that dead birds numbered approximately 100 to 150 per day, and a rising proportion of the birds expressed anorexia and stunted growth. During the same period, neighboring broiler and breeder flocks in the area reported the appearance of respiratory infection with a similar clinical display.

### 2.3. Gross Findings

**Figure 2:**
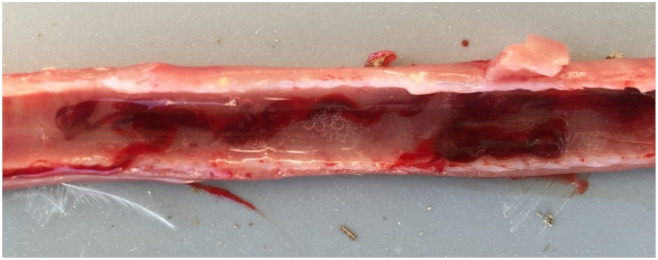
The trachea of a 33-day-old broiler chicken with typical symptoms of ILT, where hemorrhage and congestion of the trachea are visible, accompanied by bloody mucoid exudate in the lumen.

Twenty deceased and moribund birds were submitted for post-mortem examination to the Unit of Avian Medicine, Faculty of Veterinary Medicine, School of Health Sciences of the Aristotle University of Thessaloniki. The gross lesions were restricted to sinuses and the upper respiratory tract. Gross lesions included mucoid inflammation of the trachea and larynx, while in more than half of the birds, a mild to severe degree of hemorrhages, excess mucous, and blood casts were present inside the lumen of the trachea, such as the trachea appearing in Figure 2. Moreover, edema and congestion of the epithelium of the conjunctiva and the infraorbital sinuses were apparent, accompanying hemorrhage of the larynx.

### 2.4. Histopathological Examination

**Figure 3:**
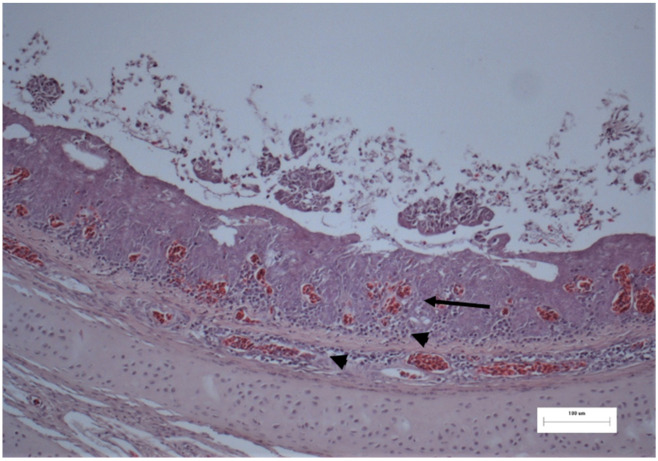
Histology of the trachea from a broiler chicken. Mucosa is thickened, epithelium is edematous, congested (arrow). Mucosa and submucosa are heavily infiltrated by lymphocytes and plasma cells (arrowhead). H.E. stain; M: 250×.

**Figure 4:**
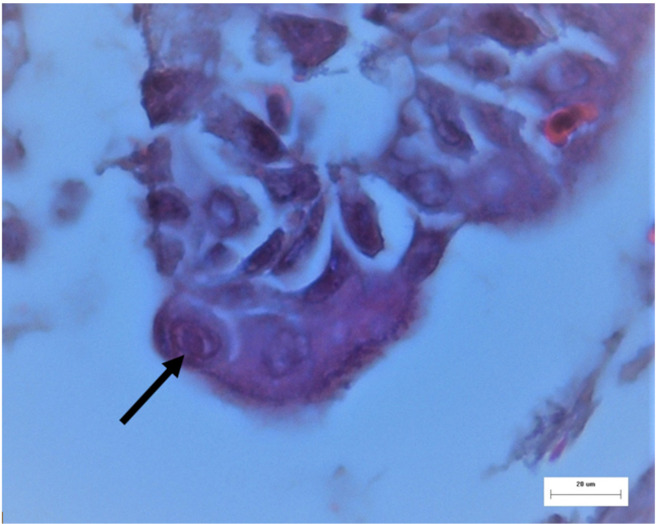
ILT, trachea from a broiler. Intranuclear viral inclusion body in an epithelial cell (arrow). H.E. stain; M: 400×.

Tissues from the trachea and conjunctiva were collected and submitted to the Scientific Support and Investigation Unit, Ceva-Phylaxia, Budapest, Hungary. They were fixated using a formaldehyde solution and embedded in paraffin. Sections were stained with the hematoxylin and eosin (H.E.) staining procedure. Epithelial hyperplasia, metaplasia, multifocal degeneration and, in a few epithelial cells, nucleic inclusion bodies were detected in the trachea (Figure 3 and Figure 4). Nevertheless, no remarkable histological lesions could be found in the conjunctiva. Unpublished data from histopathological examination in flocks located in the surrounding area likewise confirmed the presence of ILT in birds with similar clinical manifestations, strengthening the incident of horizontal spread of the virus.

### 2.5. Molecular Examination

Following the histopathological results and aiming to verify and further investigate the source of the outbreak, trachea and larynx tissues, as well as conjunctival and tracheal swabs were collected for PCR testing. DNA was extracted from the samples with the use of a QIAamp DNA Mini Kit (Qiagen, Hilden, Germany), according to the manufacturer’s recommendations. The TK gene of the ILTV was amplified with a TKOP primer set by Han and Kim [15]. Amplified products, with a length of 1092 bp, were purified and submitted for sequencing (Biomi Ltd., Gödöllő, Hungary). Sequence fragments were analyzed and aligned with MEGA 7 software, and comparison of the most similar ILT sequences was achieved with the use of the BLAST algorithm and were downloaded from the GenBank database [33]. The revealed sequences, D5203/3/4 and D5203/3/5, of 1092 nucleotides each, were 100% identical with each other, and with seven ILT field isolate sequences (Table 1). They were discriminated from the vaccine strains CEO and TCO, according to Neff et al. [16]. Therefore, we concluded that the virus strain responsible for the outbreaks in the region was possibly related to a wild type circulating strain.

### 2.6. Differential Diagnosis

At 40 days old, blood collection from the flock took place, with the serum submitted for testing against IBV, *Mycoplasma gallisepticum* (Mg), *Mycoplasma synoviae* (Ms), NDV, avian influenza (AI), *Ornithobacterium rhinothacheale* (ORT), avian rhinotracheitis virus (ART), *Avibacterium paragallinarum* and *Pasteurella multocida* antibodies, with the use of enzyme-linked immunosorbent assay (ELISA) commercial kits. Indirect ELISA tests were proven negative for AI (CK121 AI, Biochek UK Ltd., Ascot, UK), ORT (CK108 ORT, Biochek UK Ltd., Ascot, UK), ART (CK120 ART, Biochek UK Ltd., Ascot, UK), Mg and Ms ((Combined) CK215 MG/MS, Biochek UK Ltd., Ascot, UK) and *A. paragallinarum* and *P. multocida* ((Combined) AE-310905-1, Alpha Diagnostic Intl. Inc., San Antonio, Texas, USA). Correspondingly, results concerning NDV (CK116 ND, Biochek UK Ltd., Ascot, UK) and IBV (CK119 IBV, Biochek UK Ltd., Ascot, UK) were interpreted as negative, with the titer levels as expected post-vaccination of 40-day-old broilers. The youth of the flock and the results of the histopathological examination excluded the presence of the avian poxvirus.

### 2.7. Therapeutic Approach

Immediate and thorough application of control measures followed the diagnosis of ILT. A quarantine protocol and hygiene measures were applied in the infected farm, including safe disposal of the dead birds, personal sanitation procedures, and the restriction of movement to other sites of interest to the poultry industry. ILT is not treatable; therefore, the health of the birds was reinforced by the administration of multi-vitamins and electrolytes. Concerning secondary bacterial infections, an antibiotic preparation (25 mg doxycycline hyclate/kg body weight/day) was administered for 5 days via the drinking water. After a course of 10 days, the symptoms gradually appeared milder, while the majority of the birds recovered. The mortality rates gradually decreased, concluding at a percentage of 0.1% the day before slaughter. At the end of the production cycle and the depopulation of the flock, the suggested cleaning protocols called for more thorough application. To be precise, cleaning and disinfection occurred with the use of 1% Virkon™ S (LANXESS Deutschland GmbH. Kennedyplatz 1, 50569, Cologne, Germany) and 1% Sanivir Plus (BIOPLAGEN, S.L., Avda. de Castilleja de la Cuesta 26, (PIBO), 41110, Sevilla, Spain), attempting to decrease the viral load. The litter was decontaminated and composted on the farm premises [2].

## 3. Discussion

ILT is a highly contagious respiratory disease in poultry, with considerable significance since its first report in the 1920s. It has been of dominant concern for layer hens and breeder flock farmers, who rear poultry for longer periods compared to broilers, and for this reason, most commercial layers are submitted to vaccination [20]. However, increasing reports in various countries worldwide also regard outbreaks in broiler farms which has altered the concept that ILT is a disease primarily concerning adult laying birds [3,4,9,22,35]. These outbreaks had been provoked by the proximity of broiler farms to vaccinated layer hens and backyard flocks, the intensive way of poultry farming with shorter cycles of production, and biosecurity gaps [2,36].

In our case report, the broiler chickens of the organic farm displayed the clinical symptoms of ILT at 25 days of age [5], contradicting the perception that traditionally, ILT is a disease observed primarily in older birds [6] (pp. 267–271). Contemporary ILT outbreaks tend to appear more regularly in young ages because new ILTV strains emerge, chicken production has a rising expansion rate, and the poultry industry intensifies and tends to become integrative, with different types of poultry such as broilers, broiler breeders, and layers reared in proximity, as well as poultry farms with birds of various ages, making these predisposing factors for the emergence of pathogens [6] (pp. 2–13). Consequently, gaps in farm biosecurity can allow the introduction of an ILTV strain with the potential of greater transmissibility and more severe clinical manifestation [37]. Adhering to the organic farm regulations, chickens should live longer and have outdoor access, presenting the risk of contact and transmission of infectious diseases from wild birds, backyard flocks and fomites to a flock [23,38]. Furthermore, the season of the reported ILT outbreaks is in line with the seasonality of ILT outbreaks during winter months, because the virus survives longer when exposed in lower temperatures [4].

Reports from Europe, where the majority of countries implement the use of live attenuated vaccines, demonstrate that 94% of field viruses accumulated over the course of 35 years were related to vaccines [23,37,39]. With a thriving number of strains circulating, recombination can occur, in the light of reports indicating that this can lead to an increase in ILTV virulence [40]. In areas with dense poultry populations that administer a live attenuated vaccine against ILT, it is challenging to discriminate the vaccinal viruses from the circulating wild strains in clinical practice [3].

The genetic similarity between vaccinal ILTV strains and field isolates is profound, because the former originates from wild viral serotypes [20]. In this particular case, the two isolated TK gene sequences were 100% identical to the field strains mentioned in Table 1, using the Nucleotide Basic Local Alignment Search Tool (BLAST) to evaluate the gene homology [16,41]. Their comparison to known TK gene sequences isolated from commercial vaccines, including those used in layer hen farms in Greece, revealed a percent identity ranging from 99.73% to 99.91%, with the nucleotide changes presented in Table 2 [2,5,26].

Although the sequencing of a sole gene is not a definitive indication that this outbreak results from a field strain, the absolute (100%) similarity with the Italian ILTV strain 4787/80 reinforces this point of view [34]. This particular strain was isolated during an ILT outbreak in Italy in the 1980s, when live attenuated vaccines had yet to be introduced in the area [42]. As an individual fact, this is not adequate to prove the definite origin of the strain, and instead makes a strong case in favor of a field viral strain incidence. Keeping that in mind, the comparison of the isolates with the Nobilis ILT TK gene of the Serva strain, used for regional flock vaccinations, revealed their percentage identity to be 99.73%, therefore further gene sequencing is needed to make their comparison more valid. With the use of Nucleotide BLAST, a comparison between the Italian 4787/80 and the above-mentioned vaccinal strain did not reveal significant similarities, challenging the incidence of homology or possible common origin of the two strains. For the above reasons, future research needs to consider the possibility of further genome analysis and classification of ILTV strains, instead of the sole identification of the pathological factor. Methods as such include RFLP, single nucleotide polymorphism analysis by real-time PCR, and sequence analysis with the identification of target genes [15,16,26]. Apart from research purposes, this can give a clearer epidemiological profile of the disease and its possible origin on a worldwide scale [13,18].

ILTV has been characterized as highly contagious in the literature, with various means of transmission, for instance, shared poultry equipment and wind vectors [1,29]. Although the most frequent way that ILT is spread is from direct contact with respiratory exudates from acutely infected birds, the risk of indirect and/or mechanical transmission still prevails; consequently, the definite route of virus introduction can only be hypothesized [2,7]. As mentioned above, the farm under study was defined by large and very dense poultry populations and intensive poultry production, where backyard, broiler, broiler breeder, and layer flocks were neighboring, not mentioning the busy commercial poultry activity of the area. Moreover, the plain in which the poultry farm resides is an area utilized for the scattering of poultry manure from other farms of the area. All these are recognized as high-risk factors for ILT outbreaks because ILT is an airborne disease [4,35]. Therefore, the circulation and transmissibility of wild strains of ILTV, parallelly to vaccine strains, must not be undermined and kept into consideration [23].

The control measures applied, as mentioned above, consisted of the application of a quarantine protocol combined with hygiene measures and the additional supply of antibiotics, vitamins, and electrolytes. Regarding that, equally important is the fact that there is no treatment available against ILT symptoms and lesions, and to that end, a vaccination strategy must be accessible for application in the emergence of an outbreak [13]. The same applies in the emergence of Marek’s disease, another alphaherpesvirus, with its vaccine administered either in ovo or at hatch [43]. Urgent ILT vaccination programs can be initiated in field outbreaks for control and containment, with the administration of modified live vaccines, particularly the CEO vaccine with spray administration, so that unaffected birds develop and adapt immune responses against the ILTV. Nevertheless, the CEO vaccine has the downside of reversion to virulence, recombination and settlement of clinical symptoms, and growing birds potentially face those consequences. TCO vaccines are seldom used in urgent vaccination practices because the establishment of immunity is not immediate and incapable of restraining the spread of the disease to the flock, and field observations also indicate its potential to regain virulence [20]. Recombinant vaccines, even though they have the downside of not preventing the shedding of the virus and the application is not easily achieved in field conditions, have the advantage of being entirely safe, with no side effects. Their application neither introduces vaccinal ILTV strains to an area nor spreads to unvaccinated poultry and is genetically stable [22].

In our case, a recombinant vectored vaccine was administered at hatch for three consecutive production cycles, combined with the improvement of management practices and the application of strict biosecurity measures, after veterinary consultation. However, the strain responsible for the outbreak was already circulating in the area, and an industry-wide holistic approach was also applied, with the vaccination of neighboring broiler and breeder flocks with the same vaccine, the rapid molecular diagnosis of the disease, the application of stricter biosecurity protocols and by giving special attention to the feed delivery system, the crews shared between the farms, service visits, and truck routes [4]. It became apparent that there is a necessity for stricter surveillance programs and thorough research to identify and comprehensively control major animal health concerns. The outcome of this holistic effort was proven fruitful, considering that one year after the utilization of the control measures, there was a significant decrease in ILT cases in local flocks, and, to this day, no novel ILT outbreaks have been detected and reported.

To conclude, almost one century after its first report, ILT is still a disease that concerns the poultry industry, with economic losses and a worldwide dispersion of outbreaks. As far as we are aware, this is the first report of ILT in an organic broiler farm in Greece caused by a field strain of *Gallid alphaherpesvirus 1* instead of vaccinal strain. It is a necessity to modify the vaccination protocols of broiler chickens, especially for those in organic farms, because they are raised for a longer time and exposed to abundant hazard factors. The most critical issue in the success of controlling ILT in an area is the holistic approach from the entire poultry industry, which should follow an established unanimous control program. Overall cooperation to a plan might introduce complications, considering that it can potentially add to the labor and vaccine costs, but must not be discarded in every circumstance.

## Figures and Tables

**Table 1:** The list of sequences from GenBank which are identical with the Greek strain based on the TK gene sequence.

Acc. No.	Strain ID	Country	Year	Reference
MF405080	Rus/Ck/Penza/2013/2701	Russia	2013	-
MG775240	TN 41/17	Tunisia	2017	-
EU360946	CH04	Switzerland	Unknown	[16]
HM230794	288269/2007	Italy	2007	[33]
KP677883	193435/07	Italy	2007	[34]
KP677884	757/11	Italy	2007	[34]
KP677885	4787/80	Italy	2007	[34]

**Table 2:** Summarized results of the positions of nucleotide changes according to Neff et al., 2008 [16]. Dots represent positions identical to the D5203/3/4 sequence, nucleotide bases of the DNA are represented by A (adenine), C (cytosine), G (guanine) and T (thymine).

GenBank Acc. No.	Strain ID	29	428	513	540	643	650	965	1014	1025
	D5203/3/4	C	T	A	T	T	G	C	T	C
	D5203/3/5	∙	∙	∙	∙	∙	∙	∙	∙	∙
MF405080	Rus/Ck/Penza/2013/2701	∙	∙	∙	∙	∙	∙	∙	∙	∙
MG775240	TN 41/17	∙	∙	∙	∙	∙	∙	∙	∙	∙
EU360946	CH04	∙	∙	∙	∙	∙	∙	∙	∙	∙
HM230794	288269/2007	∙	∙	∙	∙	∙	∙	∙	∙	∙
KP677883	193435/07	∙	∙	∙	∙	∙	∙	∙	∙	∙
KP677884	757/11	∙	∙	∙	∙	∙	∙	∙	∙	∙
KP677885	4787/80	∙	∙	∙	∙	∙	∙	∙	∙	∙
EU360950	TCO	∙	∙	∙	C	∙	∙	∙	∙	∙
EU360949	CEO	∙	∙	∙	C	∙	∙	∙	∙	∙
JN580317	CEO_low_passage	∙	∙	∙	C	∙	∙	∙	∙	∙
JN580316	CEO_high_passage	∙	∙	∙	C	∙	∙	∙	∙	∙
JQ083494	vaccine_Laryngo_Vac	∙	∙	∙	C	∙	∙	∙	∙	∙
EU423895	SL-Laryngo-vac	∙	∙	∙	C	∙	∙	∙	C	∙
EU423888	FDL-Laryngo-vac	∙	C	T	C	∙	∙	∙	∙	∙
EU423897	SP-Trachivax	∙	∙	∙	C	∙	A	∙	∙	∙
EU423891	Isbi-Bio-Laryngo	A	∙	∙	C	∙	∙	T	∙	∙
EU423890	II-Nobilis-ILT	∙	∙	∙	C	C	∙	∙	∙	T
KP677881	Nobilis_Laringovac	∙	∙	∙	C	∙	∙	∙	∙	∙
KP677882	Poulvac_ILT	∙	∙	∙	C	∙	∙	∙	∙	∙
HQ630064	Serva	∙	∙	∙	C	∙	∙	∙	∙	∙

## Data Availability

None of the data presented were deposited in an official repository.
